# SIV infection induces aging-like alterations in cardiac cellularity and macrophage populations of rhesus macaques

**DOI:** 10.1093/jleuko/qiag069

**Published:** 2026-06-05

**Authors:** Daniel I Petkov, Summer Siddiqui, David X Liu, Carolina Allers, Peter J Didier, Woong-Ki Kim, Elizabeth S Didier, Marcelo J Kuroda

**Affiliations:** Division of Immunology, Tulane National Biomedical Research Center, Tulane University, 18703 Three Rivers Road, Covington, LA 70433, United States; Division of Microbiology, Tulane National Biomedical Research Center, Tulane University, 18703 Three Rivers Road, Covington, LA 70433,United States; Division of Comparative Pathology, Tulane National Biomedical Research Center, Tulane University, 18703 Three Rivers Road, Covington, LA 70433, United States; Division of Immunology, Tulane National Biomedical Research Center, Tulane University, 18703 Three Rivers Road, Covington, LA 70433, United States; Division of Comparative Pathology, Tulane National Biomedical Research Center, Tulane University, 18703 Three Rivers Road, Covington, LA 70433, United States; Division of Microbiology, Tulane National Biomedical Research Center, Tulane University, 18703 Three Rivers Road, Covington, LA 70433,United States; Division of Microbiology, Tulane National Biomedical Research Center, Tulane University, 18703 Three Rivers Road, Covington, LA 70433,United States; Division of Immunology, Tulane National Biomedical Research Center, Tulane University, 18703 Three Rivers Road, Covington, LA 70433, United States

**Keywords:** cardiovascular disease, comorbidities, HIV, immunohistochemistry, inflammation

## Abstract

People living with HIV experience an earlier risk for onset of aging-related chronic inflammatory comorbidities, including cardiovascular disease. We previously showed that the proportion of macrophages was higher in the heart of older rhesus macaques (*Macaca mulatta*) with normal histopathology as well as in all age groups of macaques with cardiac pathology. In the present study, we investigated the effects of simian immunodeficiency virus (SIV) infection on cell density and macrophages in heart tissues of adult rhesus macaques. Heart tissues were evaluated by hematoxylin and eosin staining and immunofluorescence staining to assess the number and phenotype of heart macrophages that could change with SIV infection. Cardiac cellularity was lower in hearts of SIV-infected young and adult macaques, as well as in uninfected aged macaques, compared with uninfected adult rhesus macaques. This lower cellularity was associated with higher percentages of CD163^+^ macrophages in hearts of SIV-infected young adult macaques, similar to that of uninfected aged macaques. Higher percentages of CD163^+^ macrophages were also observed in diseased hearts of both SIV-infected and uninfected adult animals that appeared to be short-lived macrophages, based on 5-bromo-2′-deoxyuridine labeling. In contrast, macrophages retaining in vivo–administered dextran, indicative of long-lived macrophages, predominated among cardiac macrophages in both SIV-infected and uninfected animals of all ages. Together, these results suggest SIV infection induces changes in cardiac cellularity and macrophage composition that resemble those observed during aging. This rhesus macaque model supports continued studies to further dissect the shifting dynamics of macrophage subsets and their role in heart adaptations in response to HIV infection and aging.

## Teaser

The increased percent of macrophages in the heart with decreased number of heart cells in aged macaques is also observed in young macaques with SIV infection.

## Graphical Abstract

**Graphical Abstract:**
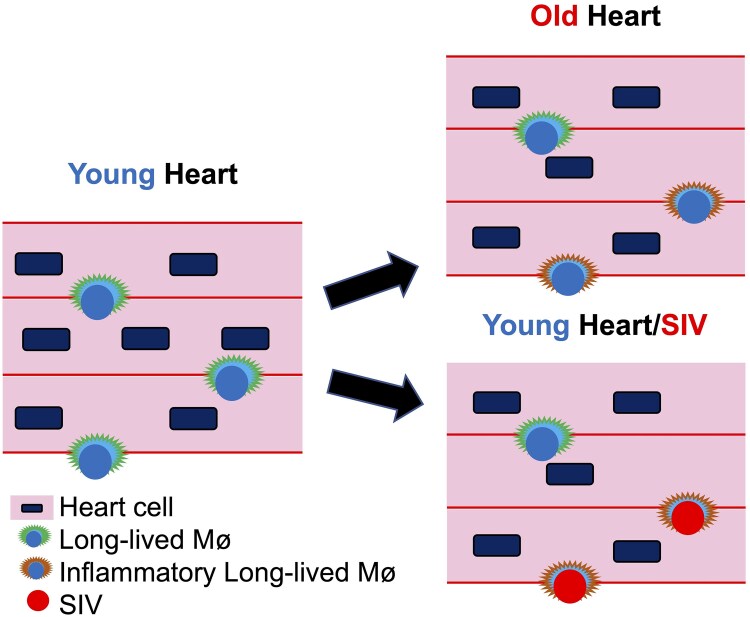

## 1. Introduction

Combination antiretroviral therapy (ART) effectively suppresses plasma viral load and prolongs survival in people living with HIV. However, these individuals experience accelerated aging and develop chronic, inflammatory, HIV-associated non-AIDS (HANA) comorbidities, including cardiovascular disease (CVD), that occur earlier than in the non–HIV-infected population.^1–9^ Currently, it is estimated that people living with HIV have nearly a 2-fold greater risk of cardiovascular-related morbidity and mortality despite ART treatment, compared with HIV-uninfected populations.^10^

The etiology of cardiac-related and other comorbidities in HIV-infected individuals is likely multifactorial. Although ART attains effective viral suppression, persistence of the virus within tissue and cellular reservoirs^9,11–14^ and incomplete recovery of immune response regulation contribute to sustained immune activation and chronic inflammation.^6,15,16^ Chronic inflammation is central to the higher risk for CVD.^6^ Consequently, increasing attention has focused on the role of macrophages in CVD because they are central regulators of inflammation and fibrosis. In addition, macrophages may be a cellular reservoir for HIV, thereby contributing to viral persistence and dysregulated inflammatory responses.^9,12,13,17,18^

Distinct populations of tissue macrophages have been described in studies using mice, nonhuman primates, and humans, among other hosts.^19,20^ Based on mouse studies, tissue macrophages comprise longer-lived resident macrophages with self-renewal properties, as well as short-lived macrophages replenished from circulating monocytes.^21^ Monocytes also have been delineated into classical, intermediate, and nonclassical subsets with unique functions in inflammation and tissue repair.^22,23^ The relative contribution of these macrophage populations shifts in response to infection, tissue injury, stress, and aging in order to re-establish homeostasis.^19^ Cardiac macrophages can be further distinguished using markers associated with differentiation state and tissue residency. CD163 is a hemoglobin–haptoglobin scavenger receptor commonly expressed by monocyte-derived macrophages and is often enriched in recently recruited or higher-turnover populations. In contrast, HAM56 and CD206 (mannose receptor) are widely used markers of more differentiated, tissue-resident macrophages in nonhuman primates and are associated with homeostatic and reparative functions within tissues.

Nonhuman primates are physiologically similar to humans and are amenable for studying cardiac macrophage biology and tissue adaptation.^24–29^ In a previous study of uninfected rhesus macaques, we compared cellularity and macrophage populations in heart tissues of pediatric, adult, and older rhesus macaques.^25^ Cardiac cellularity was higher in pediatric animals than in adult and aged animals. Among macrophages, the CD163^+^ (scavenger receptor) macrophages predominated over HAM56^+^ and CD206^+^ (mannose lectin receptor) macrophages in heart tissues. The percentage of CD163^+^ macrophages was higher in older macaques (ie older than 13 yr) with normal histopathology, as well as in heart tissues of adult animals exhibiting cardiac lesions (ie those with higher histopathology scores or disease) compared with the mean percentage of CD163^+^ macrophages in adults with low histopathology. Furthermore, the majority of cardiac macrophages in adult animals were considered longer lived, based on retention of in vivo dextran administration.

Our purpose in conducting the present study was to examine any shifts in cellularity and macrophages in hearts of rhesus macaques after simian immunodeficiency virus (SIV) infection and to determine whether these changes resemble those observed during aging. To examine macrophage subsets, turnover and longevity in heart tissues from SIV-infected animals, we aimed to better understand their potential role in cardiac adaptation during HIV infection and accelerated aging.

## 2. Materials and methods

### 2.1. Animals and inoculations with dextran, SIV/SIV-HIV, and 5-bromo-2′-deoxyuridine

Rhesus macaques (*Macaca mulatta*) of Indian origin of both sexes (*n* = 52) and ranging in age from 14 d to 24 yr were housed at the Tulane National Biomedical Research Center for the studies reported here (Table 1). At the time of assignment, all animals were specific pathogen–free of macacine herpesvirus 1, SIV, simian retrovirus, and simian T lymphotropic virus 1, as well as negative for measles virus and *Mycobacterium tuberculosis*. All procedures were performed in accordance with the National Institutes of Health *Guide for the Care and Use of Laboratory Animals*^30^ and the standards of the Association for Assessment and Accreditation of Laboratory Animal Care, and were approved by the Institutional Animal Care and Use Committee of Tulane University.

**Table 1:** Characteristics of animals used in the study.

Animal ID	Category	Age at necropsy (yr)	Sex	SIV infection	Days after infection (DPI)	Plasma viral load	Dextran administ-ration (d)	Heart pathology score	Skeletal muscle pathology score	Primary cause of death
LK36	Uninfected	0.04	M	–	–	–	–	LV-0, RV-0, RA-0	–	Undetermined
LI01	Uninfected	0.5	F	–	–	–	–	RV-0, RA-0		Lymphoid hyperplasia
KR74	Uninfected	1.9	M	–	–	–	15	LV-1, RA-0	RECTFm-0	Dilated lateral ventricles in brain
KL42	Uninfected	2.7	M	–	–	–	–	RV-0, RA-0	RECTFm-0	Colitis, splenic hyperplasia
KG16	Uninfected	2.9	M	–	–	–	15	–	RECTFm-0	Encephalomalacia, kidney disease
								LV-0, RV-0, RA-0		
K539	Uninfected	3.6	M	–	–	–	–	–	–	Myocarditis
KA51	Uninfected	3.9	M	–	–	–	256	LV-0, RA-0	RECTFm-0	Lymphoid hyperplasia
D469	Uninfected	5.5	F	–	–	–	–	LV-3		–
										Myocarditis
IP62	Uninfected	5.7	F	–	–	–	–	LV-1, RV-0, RA-0, LA-1, IVS-0, APE-1, RP-0, LP-0		–
										Chronic gastroenteritis
IB90	Uninfected	6.7	M	–	–	–	–	LV-0, RV-0, RA-4, LA-2, IVS-0, APE-0, RP-1, LP-1		–
										Chronic gastroenteritis
F442	Uninfected	10	F	–	–	–	–	LV-3		–
										Myocarditis
EL85	Uninfected	11.9	F	–	–	–	–	LV-0, RV-0, RA-0, LA-1, IVS-0, APE-1, RP-1, LP-1		–
										Chronic gastroenteritis
CE23	Uninfected	12.7	F	–	–	–	–	LV-4		–
										Myocarditis
DR52	Uninfected	13.2	F	–	–	–	–	LV-0, RA-1		–
										Chronic dermatitis
N702	Uninfected	14.7	F	–	–	–	–	LV-3		–
										Myocarditis
CE12	Uninfected	16.8	F	–	–	–	–	APE-2		Chronic suppurative ear inflammation
DB52	Uninfected	17.1	M	–	–	–	–	LV-1, RA-0		–
										Undetermined
D826	Uninfected	19.4	F	–	–	–	–	LV-3		–
										Myocarditis
P202	Uninfected	20.2	M	–	–	–	–	LV-1	–	Chronic enterocolitis, myocardial inflammation
IR91	Uninfected	22.3	F	–	–	–	–	LV-1		–
										Endometriosis
P346	Uninfected	22.8	F	–	–	–	–	LV-0		–
										Arthritis
L278	Uninfected	24	F	–	–	–	–	LV-1	RECTFm-0	–
										Endometriosis
LH06	SIV-infected	0.13	F	SIVmac251(IV)	47	1.64 × 10^7^		LV-0, RV-1, RA-4		–
										Enterocolitis
LG81	SIV-infected	0.56	F	SIVmac251(IV)	196	1.64 × 10^8^	–	LV-0, RV-0, RA-1	–	Enterocolitis, dehydration
LH34	SIV-infected	1.01	F	SIVmac251(IV)	366	1.70 × 10^7^	18	LV-0, RV-0, RA-0		–
										Undetermined
KC73	SIV-infected	3.2	M	SIVmac239(IV)	16	3.62 × 10^7^	99	LV-1, RV-0, RA-1	RECTFm-0	–
										Undetermined
H685	SIV-infected	4	M	SIVF965 (IV)	275	NA		LV-3		–
										Myocarditis
JT57	SIV-infected	4.1	M	SIVmac239(IV)	10	2.57 × 10^7^	115	LV-0, RV-0	RECTFm-0	–
										Hydrocephalus
EI59	SIV-infected	4.2	M	SIVmac239(IV)	129	NA		LV-4		–
										Myocarditis
IM24	SIV-infected	4.6	M	SIVmac239(IV)	309	7.96 × 10^3^		LV-1		Pneumonia, lymphoid hyperplasia
JL38	SIV-infected	5	M	SIVmac251(IV)	172	1.76 × 10^5^	39	LV-2, RV-4, RA-1	–	Pulmonary thrombosis
IB84	SIV-infected	5.2	M	SIVmac239(IV)	216	3.04 × 10^7^		LV-2		Lymphoid hyperplasia, dysplasia
IM90	SIV-infected	5.2	M	SIVmac239(IV)	541	2.24 × 10^6^	7	LV-1		Pneumonia, lymphoid hyperplasia
IB62	SIV-infected	5.3	M	SIVmac239(IV)	216	1.73 × 10^7^		LV-0		Pneumonia, lymphoid hyperplasia
IT57	SIV-infected	5.3	M	SIVmac239(IV)	603	3.13 × 10^5^	14	LV-1	RECTFm-1	Lymphoid hyperplasia, dysplasia
IB29	SIV-infected	5.3	M	SIVmac239(IV)	132	1.01 × 10^4^	1	LV-1		Lymphoid hyperplasia, dysplasia
IL68	SIV-infected	5.5	M	SIVmac239(IV)	624	3.60 × 10^6^	36	LV-2, RV-0, RA-0	RECTFm-0	Lymphoid hyperplasia, dysplasia
IM47	SIV-infected	5.7	F	SHIV162P3	266	1.00 × 10^2^	27	LV-2, RV-1, RA-0	RECTFm-0	Lymphoid hyperplasia,
										dysplasia
HE18	SIV-infected	6.4	M	SIVmac239(IV)	91	1.49 × 10^9^		LV-2		Pneumonia, lymphoid hyperplasia, dysplasia
IB12	SIV-infected	6.4	M	SIVmac239(IV)	631	1.13 × 10^5^	42	LV-2, RV-1, RA-1	RECTFm-2	Meningoencephalitis, lymphoid hyperplasia
AP01	SIV-infected	6.7	F	SHIV162P3	242	NA		LV-4		–
										Myocarditis
HJ16	SIV-infected	7	M	SIVmac239(IV)	390	2.34 × 10^7^		LV-1		Nephrosis, lymphoid hyperplasia
GB63	SIV-infected	9.1	F	SHIV162P3	239	1.41 × 10^6^		LV-1		Lymphoid hyperplasia,
										dysplasia
GL92	SIV-infected	9.5	M	SIVmac239(IV)	149	2.02 × 10^6^	187	LV-0, RV-1, RA-0	RECTFm-0	–
										Undetermined
FM01	SIV-infected	12.4	M	SIVmac239(IV)	82	1.64 × 10^7^	187	LV-0, RV-0, RA-0	RECTFm-0	Pneumonia, encephalitis, lymphoid depletion
AT36	SIV-infected	14	F	SIVmac239(IV)	636	NA		LV-3		–
										Myocarditis
HG45	SIV-infected	14.5	F	SIVmac251(IV)	272	NA		LV-4		–
										Myocarditis
CN60	SIV-infected	15.7	F	SHIV162P3	272	NA		LV-4		–
										Myocarditis
LF95	SIV-infected	17.8	F	SIVmac239(IV)	182	NA		RV-4		–
										Undetermined
AK07	SIV-infected	17.7	M	SIVmac239(IV)	91	1.60 × 10^8^	160	LV-1, RA-1	RECTFm-0	Pneumonia, lymphoid hyperplasia, dysplasia
P763	SIV-infected	21.9	M	SIVmac239(IV)	239	4.06 × 10^5^	157	LV-2, RA-0	RECTFm-3	Pneumonia, lymphoid hyperplasia, dysplasia
L966	SIV-infected	23.8	F	SIVmac239(IV)	118	2.95 × 10^7^	187	LV-2, RV-1, RA-2, LA-1	–	Cardiomyopathy, hydrothorax, hydropericardium

Abbreviations: DPI, days post infection; d, days; IV, intravenous; NA, not available; LV, left ventricle; RV, right ventricle; RA, right atrium; LA, left atrium; IVS, interventricular septum; APE, apex; RP, right papillary muscle; LP, left papillary muscle; RECTFm, rectus femoris muscle.

Dextran (Amino Dextran, 10,000 MW; D1860, Thermo Fisher Scientific, Waltham, MA) was administered to a subset of animals via intravenous (75 to 150 mg/kg), aerosol (100 to 300 µg/kg), and/or intrathecal (1 to 2.5 mg/kg) routes^31,32^ (Table 1). Among the animals studied, a total of 30 were inoculated with SIV_mac239_ (*n* = 19 animals), SIV_mac251_ (*n* = 6 animals), SIV_F965_ (*n* = 1 animal), or SIV-HIV_SF162P3_ (SHIV_SF162P3_) (*n* = 4 animals), as indicated in Table 1. A thymidine analog, 5-bromo-2′-deoxyuridine (BrdU) (Sigma-Aldrich, St. Louis, MO), was injected intravenously at 60 mg/kg per animal 48 h prior to necropsy. The percentage of BrdU-incorporated heart macrophages was quantified by immunostaining, as previously described.^33,34^

### 2.2. Tissue processing, histochemistry, and immunofluorescence staining

Heart tissues (namely, apex, interventricular septum, left and right atria, papillary muscles, and ventricles) and skeletal muscle (middle section of the rectus femoris) were collected at necropsy, fixed in Z-Fix (buffered zinc formalin fixative; Anatech Ltd., Battle Creek, MI), and embedded in paraffin. Longitudinal tissue sections 5-μm thick were stained with hematoxylin and eosin (H&E) using a Leica AutoStainer XL (Leica Biosystems, Buffalo Grove, IL, USA). For immunofluorescence staining, 5 μm–thick tissue sections were deparaffinized with xylene, rehydrated through graded ethanol and deionized water, and microwaved for 20 min for antigen retrieval with 0.1% Tween 20 in 10% high pH solution (Vector Laboratories, Burlingame, CA; H3301). Sections were then transferred to a preheated 2% antigen unmasking solution (Vector Laboratories, H3300) for 30 min. Autofluorescence was reduced by incubation in 0.05% Sudan Black B for 5 min. Sections were blocked with 10% normal goat serum (Thermo Fisher Scientific, 16210-064) for 40 min and incubated for 1 h at room temperature with primary Ab followed by incubation with secondary Ab for 30 min (Table S1). Abs were diluted in PBS with 0.2% cold-water-fish-skin gelatin (FSG) (Millipore-Sigma, St. Louis, MO; G-7765), and slides were washed twice for 10 min with PBS-FSG-Triton 100 (Millipore-Sigma, X-100) after each Ab incubation. Nuclei were counterstained with 0.2 μg/mL DAPI dilactate (Thermo Fisher Scientific, D3571) in PBS for 10 min at room temperature. Slides were washed and coverslipped using a fluorescence mounting medium composed of 2.4 g Mowiol 4-88 (Calbiochem, 475904), 6 g of glycerol (Millipore-Sigma, G6279), and 6 mL of double-distilled H_2_O.

#### 2.2.1. Histopathology scoring

H&E-stained heart and skeletal muscle sections were scored for lesions by 2 pathologists using high-power-field (HPF) magnification (40× objective lens or 0.09048 mm^2^), as previously described.^25,35^ Histopathology scores were defined as follows: 0 indicated normal tissue with no inflammatory infiltrate; 1, minimum, with 1 to 5 mononuclear cells/HPF or 1 to 2 foci per tissue section; 2, mild, with 6 to 20 mononuclear cells/HPF or 3 to 5 foci per tissue section; 3, moderate inflammation, with more than 20 mononuclear cells/HPF or 6 to 20 foci per tissue section; and 4, severe inflammation, with multifocal to coalescent inflammatory infiltrate. Histopathology scores of 0 to 3 were also assigned to skeletal muscle from both SIV-infected and non–SIV-infected animals (Table 1). Because normal heart and skeletal muscles consist of numerous cell types, in addition to macrophages and monocytes, distribution of macrophages in these tissues was enumerated in relation to total numbers of cells (based on numbers of cell nuclei).

### 2.3. Image analyses

Photomicrographs were taken from 11 random HPFs per tissue section that equaled to 1 mm^2^ area—that is (0.09048 mm^2^ / HPF) × 11 HPFs = 1 mm^2^—using a 40× objective of a Leica DMRE microscope (Leica Microsystems; Wetzlar, Germany) equipped with a Nuance FX camera (PerkinElmer; Waltham, MA). Images containing large blood vessels were excluded from analysis. The percentages of cells expressing each biomarker were calculated using the total number of DAPI-stained nucleated cells as the denominator. The mean fluorescence intensity of cells stained with individual fluorochromes was measured and compared using normalized values for each fluorochrome, which were calculated as counts/(gain × binning2 × exposure time × 2bit depth). Images were analyzed using InForm (PerkinElmer) and Fiji software.^36^

### 2.4. Statistical analyses

Median values and interquartile ranges (IQRs) of results were calculated for all datasets. A nonparametric Mann–Whitney test was used for comparisons between 2 groups, and the Kruskal–Wallis test followed by Dunn's post hoc tests were applied for comparing more than 2 groups. Spearman’s analysis was used for measuring correlations. A *P* value < 0.05 was considered statistically significant. All statistical analyses and graphs were prepared using GraphPad Prism, version 9.0.2 for Windows (GraphPad Software, La Jolla, CA). Venn diagrams were generated with online software (https://omics.pnl.gov/software/venn-diagram-plotter; Pacific Northwest National Laboratory, US Department of Energy) to illustrate the percentage of macrophages expressing individual biomarkers or combinations of biomarkers (ie CD163, HAM56, and/or CD206) relative to total heart cell nuclei.

## 3. Results

### 3.1. Cell density is lower in hearts of adult rhesus macaques infected with SIV compared with uninfected controls

**Figure 1:**
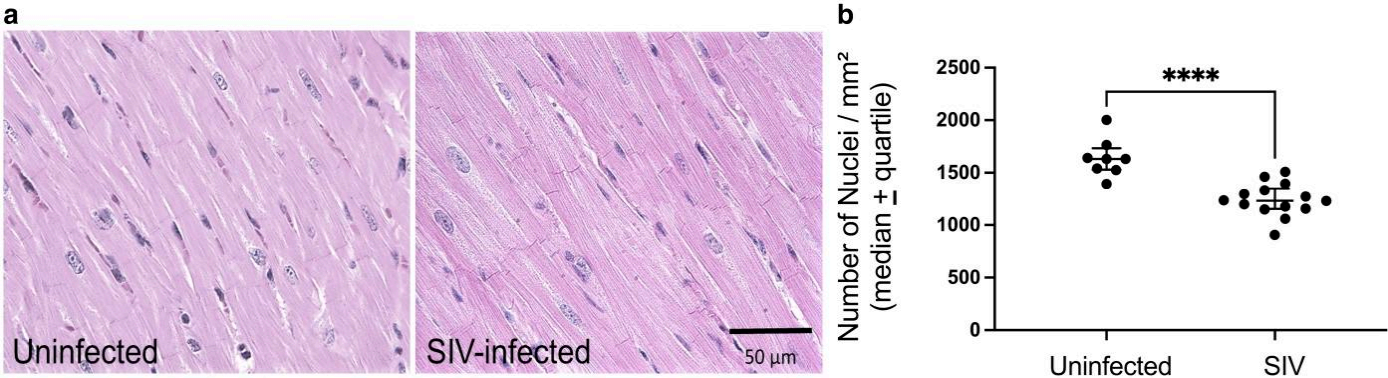
Lower cell density in adult SIV-infected rhesus macaques. (a) Representative images of H&E-stained tissue sections of the left ventricle from an uninfected rhesus macaque aged 12 yr (animal EL85; left photo) and the right ventricle of a 6-yr-old rhesus macaque infected with SIV (animal IB12; right photo). Images were acquired at 40x maginification, for quantification of H&E staining, 1 section for each rhesus macaque was analyzed to obtain the summary. (b) The number of cell nuclei per mm^2^ were counted in DAPI-stained tissue sections from left or right ventricles of uninfected (*n* = 8; 2 to 12 yr), and SIV-infected (*n* = 14; 4 to 12 yr) rhesus macaques. Histopathology scores ranged from 0 (normal) to 2 (mild), as described previously.^25^ Statistical comparisons were performed using a Mann–Whitney test; ****P < 0.0001.

We first compared the cellularity in heart tissues of SIV-infected rhesus macaques (juveniles to adults, age range: 2 to 14.5 yr) with that of age-matched uninfected control macaques. Based on H&E staining, overall cellularity was lower in the hearts of SIV-infected than uninfected rhesus macaques (Fig. 1a). To quantify these differences, the numbers of cell nuclei per area (mm^2^) in heart tissues were enumerated after DAPI nuclear staining and compared between groups of uninfected and SIV-infected rhesus macaques (Fig. 1b). A Mann–Whitney test demonstrated a significant difference between the groups (*P*  *<* 0.0001). There were lower median numbers of cells (nuclei)/mm^2^ in SIV-infected animals (1,233 cells/mm^2^; IQR 1,155 to 1,348) compared with uninfected animals (1,632 cells/mm^2^; IQR 1,529 to 1,734).

### 3.2. Numbers and percentages of CD163^+^ macrophages in hearts of SIV-infected rhesus macaques are higher than in uninfected macaques

HIV infection has been associated with increased inflammation and accelerated aging,^7,8,37^ along with higher proportions of macrophage that coincided with lower cellularity in heart tissues of aging rhesus macaques.^25^ To evaluate lymphocyte populations in this context, ventricular tissue sections from adult rhesus macaques with histopathology scores of 0 to 2 were stained for DAPI, CD3^+^ T lymphocytes, and CD20^+^ B lymphocytes and examined by fluorescence microscopy (Fig. S1a). Although not statistically significant by Mann–Whitney *U* test, SIV-infected animals had a higher median percentage of CD3^+^ T cells compared with uninfected control animals (Fig. S1b).

**Figure 2:**
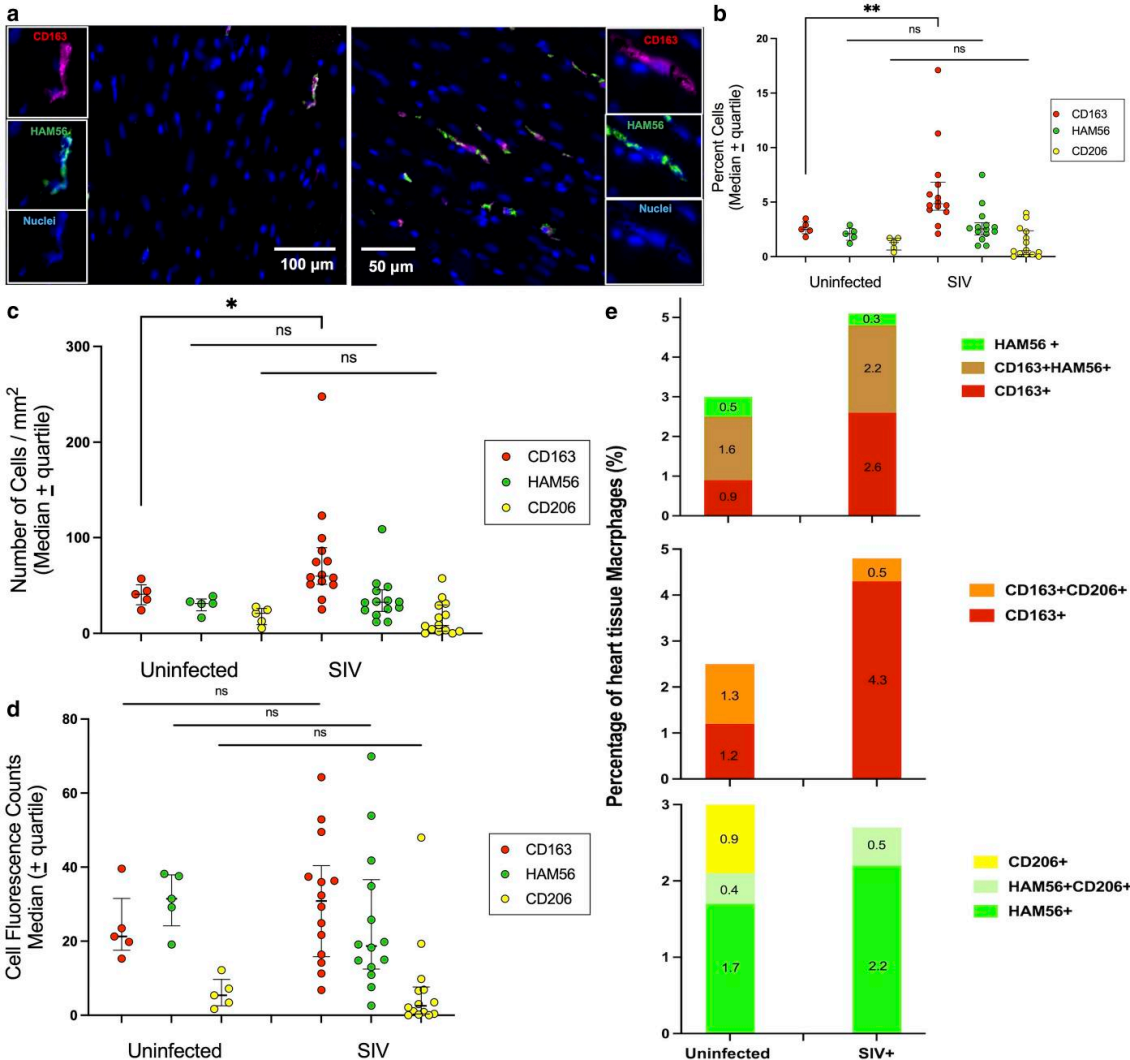
Comparison of macrophage populations in rhesus macaques without and with SIV infection. (a) Representative immunofluorescence images (40x) of right ventricle heart tissues demonstrating macrophages that expressed CD163 (red; animal KL42; uninfected, left panel) and HAM56 (green; animal KC73; SIV-infected, right panel). DAPI-stained cell nuclei are shown in blue. One section for each rhesus macaque was analyzed to obtain the summary. (b) Ventricle tissues from adult animals of aged 2 to 14.5 yr that exhibited histopathology scores of 0 to 2 (normal to mild) were stained for CD163, HAM56, and CD206, as well as DAPI, to count nucleated cells. Percentages of macrophages per total nucleated cells were counted in rhesus macaques that were uninfected or infected with SIV. (c) The numbers of macrophage subsets/mm^2^ tissue area were counted and compared between groups of rhesus macaques. (d) Cell fluorescence intensity of the stained macrophages was compared. Mann–Whitney *U* test analyses were performed on results shown in (b) (*P* = 0.0046) and (c) (*P* = 0.0258). (e) Stacked bar charts illustrate the expression of macrophages biomarkers in pair sets. Numbers within the bars indicate the percentages of each individual macrophage population.

We also examined whether chronic SIV infection altered macrophages in heart tissue of macaques aged 2 to 14.5 yr compared with uninfected macaques. Ventricles of heart tissues with normal-to-mild histopathology (score 0 to 2) were stained for the macrophage markers, CD163, HAM56, and CD206, along with DAPI, to detect cell nuclei (Fig. 2a). There was a statistically significant difference in the percentages of CD163^+^ macrophages in heart tissues between the 2 groups of animals (*P* = 0.0046) (Fig. 2b). The median percentage of CD163^+^ macrophages in the ventricles was higher in the SIV-infected animals (4.85%; IQR 4.25 to 6.825) compared with uninfected animals (2.5%; IQR 2.1 to 3.2). Percentages of HAM56^+^ and CD206^+^ macrophage subsets were not significantly different between groups.

The numbers of CD163^+^ macrophages (cells/mm^2^) significantly increased in SIV-infected animals relative to uninfected control animals (*P* = 0.0258) (Fig. 2c). Their cell fluorescence levels (Fig. 2d) were higher in the ventricles of SIV-infected adult animals but did not reach statistical significance. In contrast, no significant differences were observed in the numbers of HAM56^+^ and CD206^+^ heart tissue macrophages between SIV-infected and uninfected animals.

Co-expression of macrophage biomarkers for CD163, HAM56, and CD206 were also measured in the heart tissues of uninfected and SIV-infected groups of adult rhesus macaques (Fig. 2e). The majority of macrophages expressed CD163, and 64% of these (ie 1.6% of double-stained cells of 2.5% total CD163 positive cells) also stained positive for HAM56 in the uninfected monkey heart tissue. This proportion declined to 45.8% (ie 2.2% of double-stained cells of 4.8%) of CD163^+^cells that co-stained for HAM56 in the SIV-infected animals. A similar trend was observed among the CD163^+^ macrophages co-staining with CD206. In heart tissues of uninfected animals, 52% of CD163^+^ cells (or 1.3% of 2.5% CD163-staining macrophages) were positive for CD206. This proportion reduced to 11.4% of CD163^+^ cells (or 0.5% of 4.8% total CD163+ stained cells ) that were positive for CD206 in the SIV-infected monkey heart tissue. The percentage of HAM56^+^ cells that co-expressed CD206 was 19% (0.4% of 2.1% total HAM56 - stained cells) in the heart tissues of uninfected animals, and 19% (or 0.5% of 2.7% of HAM56^+^ cells) in SIV-infected animals. Notably, nearly all CD206^+^ macrophages co-stained for HAM56^+^ in heart tissues of SIV-infected adult (Fig. 2e).

### 3.3. Long-lived macrophages predominate in heart tissues of SIV-infected and uninfected rhesus macaques

**Figure 3:**
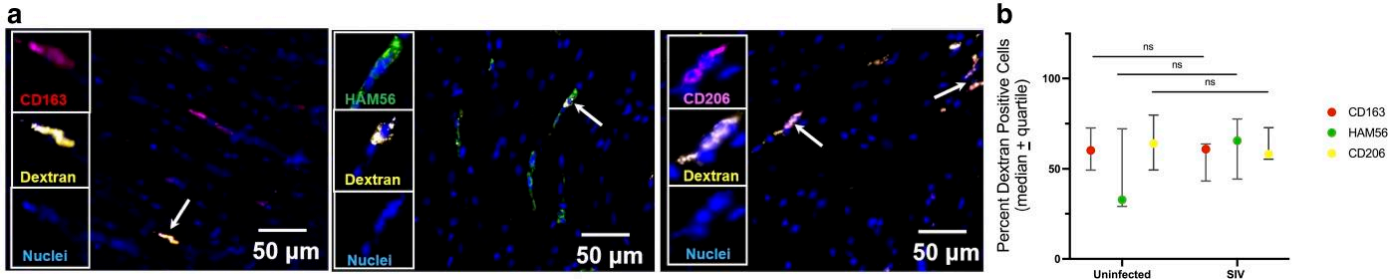
The majority of macrophages in heart tissues were long-lived based on retaining in vivo–administered dextran in uninfected, SIV-infected, adult rhesus macaques. (a) Representative images of ventricle tissues from rhesus macaques show immunofluorescent staining of macrophages retaining dextran (yellow) and expressing CD163 (red on left image; animal JT57), HAM56 (green, middle image; animal JT57), and CD206 (red, right image, animal KC73). DAPI (blue) was used to stain cell nuclei. One section for each rhesus macaque used for quantification of immunofluorescence staining. All the images were taken at 40x maginification. (b) The percentages of macrophage subpopulations with dextran were counted in heart tissues with normal-to-mild (0 to 2) histopathology of uninfected (*n* = 3), and SIV-infected (*n* = 8) adult rhesus macaques. Comparisons were measured by Mann–Whitney *U* test for each subset and there were no statistically significant differences.

Long-lived macrophages incorporate and retain in vivo–injected dextran that can be detected weeks to months later, whereas shorter-lived macrophages containing dextran die after a few days of uptake, with a half-life of approximately 2 d.^38^ Ventricles were obtained 93 to 115 d after dextran administration from 3 uninfected control rhesus macaques (aged 1.9 to 5.7 yr) and 8 chronic SIV-infected macaques (aged 5 to 23.8 yr) (Table 1). All heart tissues examined had low histopathological scores of 0 to 2 and were stained for expression of subset of markers CD163, HAM56, and CD206, as well as for dextran, to detect long-lived macrophages and DAPI to identify cell nuclei (Fig. 3a). Although there was a significantly higher percentage of CD163^+^ macrophages in heart tissues of SIV-infected animals compared with uninfected animals (Fig. 2b), the percentages of CD163^+^, dextran-retaining, long-lived macrophages as well as those expressing HAM56 or CD206 did not differ significantly between the groups of animals (Fig. 3b). However, these results demonstrated that most cardiac macrophages retained dextran and, therefore, were considered long-lived macrophages that predominate in the hearts regardless of SIV infection.

### 3.4. Lower cellularity and higher percentages of CD163^+^ macrophages in heart tissues of SIV-infected adult macaques and uninfected older macaques

**Figure 4:**
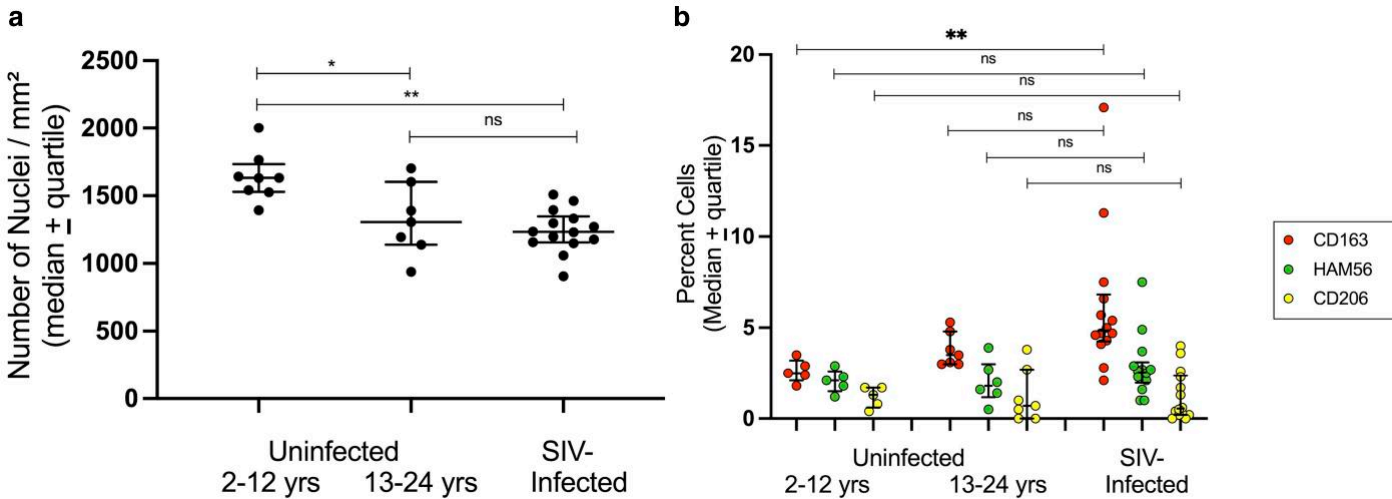
Lower cellularity and higher CD163^+^ macrophage percentage in hearts of older uninfected animals and in younger adult animals infected with SIV. (a) The number of cell nuclei/mm^2^ were counted in ventricles of rhesus macaques with histopathology scores of 0 to 2 (normal to mild). SIV-infected animals ranged in age from 4 to 12 yr. Comparisons were measured by Kruskal–Wallis test (*P* = 0.0016) and Dunn's pairwise post hoc test was applied. *P < 0.05, **P < 0.001. (b) Immunohistochemistry was applied to ventricle tissues for counting macrophages expressing CD163, HAM56, and CD206; cell nuclei were detected by DAPI staining. For quantification analysis, only 1 section was used for each animal. Comparisons for each set of macrophages were measured by Kruskal–Wallis test, which was statistically significant for CD163^+^ macrophages (*P* = 0.0067). Dunn's pairwise post test was then performed for this subset of cells. **P < 0.001.

Next, we compared cellularity in heart tissues of SIV-infected adult macaques relative to uninfected adult macaques and uninfected aged macaques with normal-to-mild histopathology. There was a significant difference in cellularity between the groups (*P* = 0.0016) (Fig. 4a). Pairwise post-test analyses indicated that cellularity was significantly lower in the heart tissues of uninfected aged animals (aged 13 to 24 yr; *P* < 0.05) as well as in SIV-infected adult animals (aged 4 to 12 yr; *P* < 0.001) compared with younger uninfected adult macaques (aged 2 to 12 yr). Importantly, cellularity did not differ significantly between uninfected aged animals and SIV-infected adult animals, indicating that chronic SIV infection in adult macaques is associated with a cardiac cellularity profile resembling that observed during aging.

The percentages of macrophage subsets were also compared between heart tissues of uninfected younger adults, uninfected older macaques, and chronically SIV-infected adult macaques (Fig. 4b). There was a statistically significant difference in CD163^+^ macrophages between these groups of animals (*P* = 0.0067). By pairwise post-test analyses, the median percentages of CD163^+^ macrophages were significantly higher in the heart tissues of SIV-infected adult macaques (*P* < 0.001) compared with uninfected younger adults. The percentage of CD163^+^ macrophages in heart tissues of the uninfected older animals was same as that observed in SIV-infected younger adults. No significant differences were detected in percentages of HAM56^+^ or CD206^+^ macrophages in heart tissues among these groups of animals.

### 3.5. Percentage of BrdU^+^ CD163^+^ macrophages is increased in heart of SIV-infected macaques with elevated monocyte turnover

**Figure 5:**
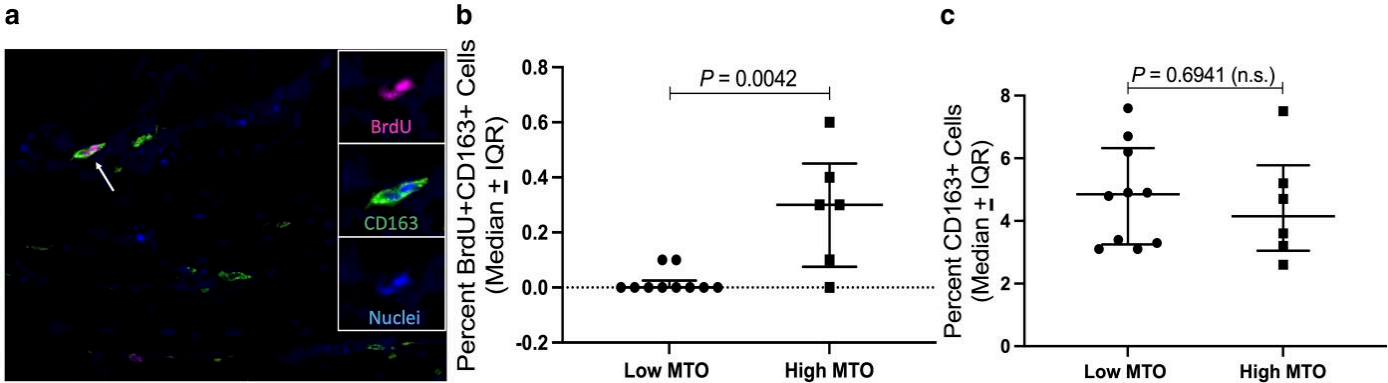
Increased trafficking of BrdU^+^ CD163^+^ monocytes/macrophages to heart tissues in SIV-infected rhesus macaques exhibiting increased monocyte turnover (MTO). SIV-infected rhesus macaques with baseline (low or < 13.2%) or increased (high or ≥ 13.2%) MTO were administered BrdU and necropsied 48 h later. Heart tissues (ventricles) were examined after immunohistochemistry. (a) An immunofluorescence image (40x) demonstrates presence of recently dividing BrdU-labeled CD163^+^ macrophages. For quantification of immunohistochemistry staining and immunofluorescence analysis, 1 section for each rhesus macaque was analyzed to obtain the summary. (b) Data plots show the percentages of BrdU^+^CD163^+^ (ie recently dividing and recruited) macrophages among total CD163^+^ macrophages in ventricles of SIV-infected macaques with high MTO compared with low MTO. (c) Results display the total percentages of CD163^+^ macrophages among nucleated cells in heart tissues of SIV-infected animals with low vs high MTO. Comparisons in (b) and (c) were performed using the Mann–Whitney test.

We previously reported that increased peripheral blood monocyte turnover (≥13.2%), in addition to CD4^+^ T-cell decline, predicts terminal disease progression in SIV-infected rhesus macaques and reflects compensatory replacement of damaged tissue macrophages, such as in the lung.^39–42^ Monocyte turnover values used in the present study were derived from the previously established peripheral blood measurements obtained longitudinally prior to necropsy. To determine whether monocyte turnover similarly reflects macrophage trafficking to heart tissues, chronically SIV-infected animals were grouped based on high (>13.2%) or low (<13.2%) peripheral monocyte turnover and administered BrdU 48 h prior to scheduled necropsy. Ventricle tissues were immunostained and examined by fluorescence microscopy to detect CD163^+^ macrophages with BrdU incorporation, which represented recently dividing and trafficking cells (Fig. 5a).

The median percentage of BrdU^+^ CD163^+^ macrophages was significantly higher in the hearts of macaques with higher monocyte turnover than in those with lower monocyte turnover (*P* = 0.0042) (Fig. 5b). In contrast, the overall percentage of CD163^+^ macrophages did not differ between SIV-infected animals with higher vs lower monocyte turnover (Fig. 5c). These findings suggest that increased BrdU^+^ CD163^+^ macrophages, as enumerated in Fig. 5b, were circulatory monocytes that had differentiated and trafficked to replace cardiac macrophages.

### 3.6. CD163^+^ macrophage population was increased in heart with more severe histopathology in uninfected and SIV-infected animals

**Figure 6:**
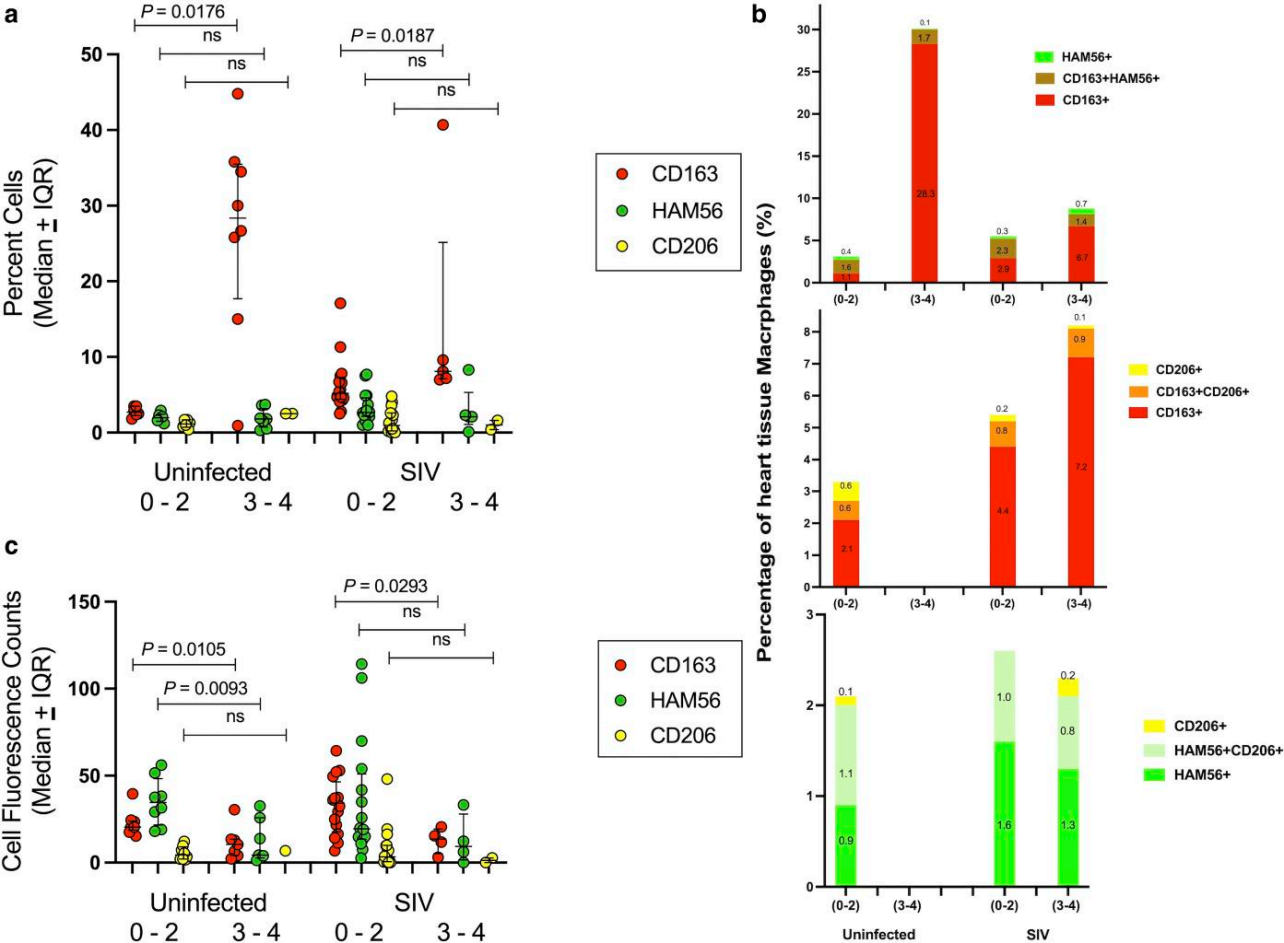
Higher percentages and lower fluorescence intensities of CD163-expressing macrophages in heart tissues with higher histopathology scores in uninfected and SIV-infected rhesus macaques. (a) Heart tissues from uninfected and SIV-infected juvenile and adult rhesus macaques with normal-to-mild (0 to 2) or moderate-to-severe (3 to 4) histopathology scores were stained for cells expressing CD163, HAM56, and CD206 and presented as percentages of cell nuclei. (b) Stacked bars illustrate the expression of macrophages biomarkers in pair sets. Numbers on the top left and lower right indicate total percentages of each individual macrophage population and numbers inside the Venn diagram indicate the percentages of single and overlapping stained macrophages. (c) Fluorescence intensity counts were plotted for cells expressing CD163, HAM56, and CD206. Mann–Whitney test was performed between results from tissues with lower (0 to 2) and higher (3 to 4) histopathology scores of uninfected animals and the SIV-infected animals shown in (a) and (c). For quantification of immunofluorescence analysis, 1 section for each rhesus macaque was analyzed to obtain the summary.

Macrophage subsets were evaluated in ventricles of rhesus macaques aged 2 to 14 yr that were uninfected or infected with SIV and compared between those with normal-to-mild (0 to 2) vs severe (3 to 4) histopathology scores (Fig. 6). In uninfected animals, hearts with higher histopathology scores had a significantly higher population of CD163^+^ macrophages than those with lower histopathology scores (*P* = 0.0016, Mann–Whitney test) (Fig. 6a). Similarly, SIV-infected macaques with higher histopathology scores had a significantly increased percentage of CD163^+^ macrophages in heart tissues compared with hearts with lower histopathology scores (*P* = 0.0187). Stacked bars

displayed proportions of dual-stained macrophages that indicated a reduction in double-positive CD163^+^HAM56^+^ cells in ventricles with more severe histopathology (Fig. 6b). In ventricles of uninfected animals, 59% of CD163^+^ cells (1.6% of 2.7% total) also were stained with HAM56 in hearts with low histopathology, compared with 5.7% of CD163^+^ cells (1.7% of 30% total) co-expressing HAM56 in hearts with higher histopathology scores. In heart tissues of SIV-infected animals, 44.2% of CD163^+^ macrophages (2.3% of 5.2%) co-expressed HAM56 with lower histopathology scores, declining to 17.3% (1.4% of 8.1% total) in hearts with higher histopathology. The median percentages of CD163^+^ macrophages that also expressed CD206 were 22% (0.6% of 2.7%) in uninfected animals, and 15.4% (0.8% of 5.2%) in SIV-infected animals with low histopathology scores; and declining further to 11.1% (0.9% of 8.1%) of CD163^+^ macrophages co-expressing CD206 in heart tissues of SIV-infected animals with severe histopathology. Among HAM56^+^ macrophages co-expressing CD206, 55.0% (1.1% of 2.0%) and 38.5% (1.0% of 2.6%) were in heart tissues of uninfected and SIV-infected macaques with low histopathology scores, respectively, and remained similar at 38.1% (0.8% of 2.1%) in heart tissues of SIV-infected macaques with higher histopathology scores.

Conversely, the percentages of single-positive CD163^+^ cells were higher in heart tissues with more severe histopathology scores. Among heart tissues from uninfected adults, single-staining CD163^+^ macrophages increased from 40.7% (1.1% of 2.7%) in heart tissues with low (0 to 2) histopathology scores to 94.3% (28.3% of 30.0%) in tissues with high (3 to 4) histopathology scores. A similar trend was observed in SIV-infected animals: the median percentage of single-positive CD163^+^ macrophages increased from 55.8% (2.9% of 5.2%) in heart tissues with low histopathology scores to 82.3% (6.7% of 8.1%) in heart tissues of animals with higher histopathology scores. This suggested that the macrophages in heart tissues with more severe disease are predominantly CD163^+^ macrophages that might be less differentiated.

We also examined the fluorescence intensities levels of macrophage biomarkers. The percentages of CD163^+^ and HAM56^+^ macrophages were higher in heart tissues with more severe histopathology scores. These cells had lower fluorescent intensity levels, indicating lower expression of these biomarkers (Fig. 6c). Specifically, CD163 expression had lower cellular fluorescence intensity in heart tissues with higher histopathology scores in both uninfected (*P* = 0.0105) and SIV-infected (*P* = 0.0293) animals compared with those with lower histopathology scores in each group, further supporting the presence of less mature or differentiating cells in heart tissues with higher histopathology scores. There was also a decline in HAM56 fluorescence intensity in heart tissues of uninfected macaques with lower vs higher histopathology scores (*P* = 0.0093), with a non-significant trend in heart tissues of SIV-infected animals.

### 3.7. Skeletal muscle macrophage subsets after SIV infection

**Figure 7:**
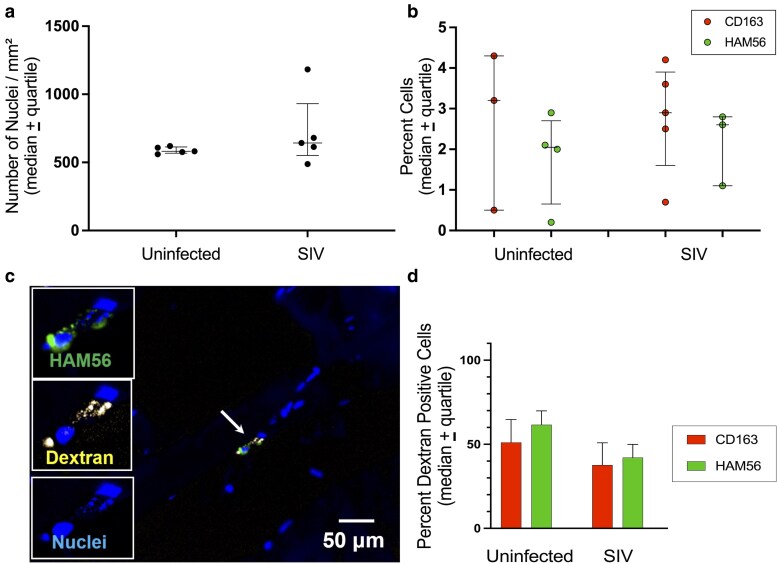
Skeletal muscle cellularity, macrophage percentages, and dextran-retaining macrophages did not differ between uninfected and SIV-infected rhesus macaques. (a) Skeletal muscle tissue sections with normal-to-mild histopathology (0 to 2) from uninfected macaques ranging in age from 3 to 12 yr and SIV-infected animals aged 4 to 12 yr were evaluated for cellularity (ie nuclei/mm^2^). (b) Immunohistochemistry was performed on skeletal tissues to determine the percentages of macrophages expressing CD163, HAM56, and CD206 relative to host cell nuclei based on DAPI staining. A Mann–Whitney test was performed to compare groups described in each panel. (c) A representative image (40x magnification) demonstrates immunostaining of skeletal muscle macrophages retaining dextran (yellow and indicated by white arrows) and HAM 56 (green). Host cell nuclei were stained with DAPI (blue). (d) The percentages of macrophage subpopulations with dextran from hearts tissues from uninfected (*n* = 5) and SIV-infected (*n* = 5) adult rhesus macaques were counted and plotted. There were no significant differences in the percentages of dextran-retaining macrophage populations in heart tissues between groups of animals (Mann–Whitney test).

Cellularity and macrophages were measured to determine if skeletal muscle reflects or simulates heart muscle dynamics after SIV infection. In uninfected adult rhesus macaques, the number of cells (nuclei)/mm2 was lower in skeletal muscle (582 cells/mm^2^; IQR 568 to 614) (Fig. 7a and 7b, respectively) than in heart tissue (1,632 cells/mm^2^; IQR 1,529 to 1,734). However, skeletal muscle cellularity did not differ significantly among uninfected animals and SIV-infected animals (Fig. 7a). The percentages of CD163^+^ macrophages remained similar between heart tissue (2.5%; IQR 2.1 to 3.2) (Fig. 2b) and skeletal muscle (3.2%; IQR 0.5 to 4.3) (Fig. 7b). Although SIV infection was associated with increased proportions of CD163^+^ macrophages in heart tissue, no significant differences were observed in skeletal muscle macrophage proportions among uninfected and SIV-infected animals. Percentages of HAM56-expressing macrophages were also similar in skeletal muscle of all groups (Fig. 7b). Approximately half of the skeletal muscle macrophages retained dextran, and no differences were observed in percentages of dextran-retaining, long-lived CD163^+^ and HAM56^+^ macrophages among the groups of animals (Fig. 7c and d), similar to results observed in heart tissue (Fig. 3b).

## 4. Discussion

Nonhuman primates are genetically and physiologically closely related to humans, and studies of HIV infection have relied heavily on the model of SIV-infected rhesus macaques, which exhibit a similar course of disease progression.^43–45^ In the absence of ART, rhesus macaques typically undergo an acute phase of infection followed by gradual CD4 T-cell decline, increased monocyte turnover, higher plasma virus levels, and development of opportunistic infections with progression to AIDS and then death. ART administration to SIV-infected rhesus macaques extended survival times, as observed in humans, and these animals similarly develop HANA conditions or comorbid conditions.^46^ HANA conditions resemble chronic inflammatory diseases that occur at an earlier age than in non–SHIV-infected hosts,^37,47^ a process considered indicative of accelerated or accentuated aging.^24,26,27,48–52^

The purpose of this study was to extend earlier findings about heart-tissue histology in uninfected pediatric, adult, and aged rhesus macaques and evaluate effects of SIV infection on heart histology in adult rhesus macaques in comparison with uninfected older macaques. Heart tissue comprises cardiomyocytes, pericytes, fibroblasts, endothelial cells, and immune cells, among which macrophages predominate.^53^ We previously reported that cellularity (cell density) was higher in histologically normal heart tissues of uninfected rhesus macaques less than 1 yr old compared with cellularity of heart tissues in groups of animals aged 2 to 12 yr and 12 to 24 yr.^25^ In this study, we detected lower cellularity in heart tissues of young adult animals (ranging in age from 4 to 12 yr) with chronic SIV infection, compared with age-matched uninfected adults. This reduction was comparable to lower cellularity observed in uninfected older animals (aged 13 to 24 yr).^25^ These results suggest that chronic SIV infection may have affected heart tissue cellularity in adult animals in a manner similar to aging. The change in heart tissue cellularity may represent an adaptive response, similar to findings in murine studies in which cardiomyocyte size was found to increase with corresponding lower cellularity during hypertension and ischemia.^54,55^

Macrophages constitute the largest immune cell population in the heart and affect adaptive cardiac remodeling during aging and disease.^54–57^ When comparing heart tissues with normal-to-mild (0 to 2) histopathology scores, we previously observed significantly higher percentage of CD163^+^ macrophages in the older animals (aged 13 yr or older) compared with heart tissues of uninfected young adult macaques (4 to 12 yr old).^25^ Furthermore, in heart tissues of young adult animals ranging from 3.6 to 19.4 yr old with moderate-to-severe histopathology scores (eg lesions with inflammatory cell infiltrates, myocardial degeneration), the percentages of CD163^+^ macrophages were significantly increased than in heart tissues with normal-mild histopathology scores in the same age range of animals. In the present study, similarly higher percentages of CD163^+^ macrophages were detected in heart tissues of SIV-infected young adult animals, comparable to uninfected older rhesus macaques, further supporting a role in SIV infection, promoting macrophage dynamics similar to those occurring in aging.

Tissue-resident macrophages in healthy rhesus macaques are phenotypically and functionally diverse^58^ and change phenotypically during aging and HIV infection.^24^ For example, the proportions of nonclassical (CD14^+^CD16^++^) and intermediate (CD14^++^CD16^+^) monocytes/macrophages are increased in humans with HIV infection or acute coronary syndrome,^59^ as well as in SIV-infected macaques that also exhibited upregulated CD68^+^ and CD163^+^ macrophages in the myocardium.^51,60^ In earlier studies of lung, we characterized shorter-lived macrophages in lung that exhibited a half-life of approximately 1 to 2 d, were relatively smaller, expressed CD163 but not CD206, and were confined primarily to the interstitial tissues of the lung.^31^ We also identified CD163^+^ and CD206^+^ longer-lived lung macrophages that were relatively larger that retained dextran for at least weeks to months and were predominantly located in the alveolar spaces. Interestingly, in the intestine, the distinctions between shorter- and longer-lived macrophages were based primarily on location and did not rely solely on surface marker expression, as seen in the lung.^34^ Specifically, intestinal short-lived macrophages were located in the lamina propria and expressed both CD163 and CD206. During SIV infection, these lamina propria macrophages were killed by the virus and replaced by trafficking less-mature CD163^+^CD206^−^ monocyte/macrophages. In contrast, longer-lived macrophages of the intestine remained in the submucosa and expressed both CD163 and CD206.

Here we examined macrophages expressing CD163, HAM56, and CD206 in heart tissues of SIV-infected rhesus macaques. CD163^+^ macrophages predominated in heart tissue of SIV-infected macaques and were present at significantly higher percentages than in hearts of uninfected macaques. The percentages of individually stained HAM56^+^ and CD206^+^ macrophages were not significantly different in heart tissues of uninfected compared with infected animals. Based on measures of dual-staining populations depicted in the Venn diagrams, the overwhelming proportion of HAM56 macrophages and CD206 macrophages co-stained for CD163. Also, in the SIV-infected animals, virtually all CD206 macrophages in heart tissues co-stained for HAM56, but in the uninfected animals, the co-staining of HAM56 and CD206 was minimal, suggesting a shift in macrophage biomarker expression after SIV infection.

Interestingly, there was a higher percentage of macrophages expressing only CD163 (without co-expression of HAM56) in heart tissues with higher histopathology scores from adult animals with or without SIV infection, as described here, as well as in pediatric and aged animals infected with SIV with normal-to-mild histopathology scores (unpublished observation). This may reflect the recruitment of less-mature macrophages to heart tissue based on the reduced fluorescence intensity of CD163 and HAM56 in heart tissues of uninfected and SIV-infected adult animals with higher histopathology scores. Further evidence for greater macrophage trafficking to hearts are provided by earlier findings that an increase in monocyte turnover of greater than 13.2% was predictive for onset of terminal disease progression in SIV-infected rhesus macaques,^41,42^ which appeared to reflect an attempt to replace damaged tissue macrophages, as measured in the lung.^39,40^ Consistent with this observation in lung, heart tissues of SIV-infected macaques with increased monocyte turnover contained higher percentages of BrdU-staining CD163^+^ macrophages than heart tissues of SIV-infected animals with lower monocyte turnover. The total percentages of CD163^+^ macrophages did not differ between SIV-infected animals with baseline and increased monocyte turnover rates, suggesting that the recently dividing BrdU-positive macrophages had migrated to heart tissues of the infected animals with increased monocyte turnover to replace some of the CD163^+^ tissue macrophages. Taken together, the higher percentage of macrophages with single CD163 biomarker expression, the lower cell fluorescence intensity in heart tissues with higher histopathology, and the higher rate of BrdU^+^ CD163^+^ macrophages during SIV infection at the stage of high monocyte turnover with impending terminal disease support an increased immigration or trafficking of macrophages to heart tissues during disease progression.

We also found that the majority of the macrophage populations expressing CD163, HAM56, and CD206 also retained dextran and thus represented longer-lived macrophages in heart tissues of SIV-infected and uninfected animals. The dextran-containing long-lived macrophages were distributed throughout the heart tissue, unlike in lung and intestine where the long-lived macrophages were primarily located in the alveolar spaces and submucosa, respectively. Previous studies indicated a preferential destruction of short-lived macrophages and survival of long-lived infected macrophages in lungs during SIV infection.^39^ The maintenance of long-lived macrophages in heart tissues of macaques during SIV infection would support the potential for virus persistence in these macrophages. It is not feasible to do repeated cardiac biopsy after cycles of BrdU administration, so additional approaches and identification of biomarkers are needed to further delineate short-lived and long-lived macrophages and their effects on adaptive responses in the heart.

In summary, results from previous studies showed that heart tissues in rhesus macaques underwent shifts such as lower cellularity in aged animals along with increased percentages of macrophages in heart tissues of all rhesus macaques with higher histopathology scores, as well as in older uninfected animals older than 19 yr with normal-to-mild heart tissue histopathology scores.^25^ The results here further examining heart tissues in young adult rhesus macaques infected with SIV similarly demonstrate an increase in heart tissue macrophages analogous to the increase in macrophages reported in heart tissues of uninfected older macaques. The current findings provide a foundation for future studies to assess the mechanisms of macrophage population shift in response to SIV infection and accelerated or natural aging, and how these affect heart function adaptations. Observations that long-lived macrophages of lung survived SIV infection^39^ and in heart are the predominant macrophage subset provide the rationale to test whether the long-lived macrophages of the heart and in other tissues contribute to the virus reservoir, become dysregulated to promote chronic inflammation, and should be targeted for modulation or replacement.

## Supplementary Material

qiag069_Supplementary_Data

## Data Availability

The datasets generated and analyzed during this study will be available from the corresponding author upon reasonable request.
