# Supplementary material for: SIV infection induces aging-like alterations in cardiac cellularity and macrophage populations of rhesus macaques
Source: J Leukoc Biol. 2026 Jun 5;118(6):qiag069. doi: 10.1093/jleuko/qiag069 (PMC13316664; doi:10.1093/jleuko/qiag069)
Supplement: qiag069_Supplementary_Data [file qiag069_supplementary_data.zip › Petkov et al Supplemental Table 1-antibodies.docx]

**Supplemental Table 1. Antibodies used in the studies.**

**_______________________________________________________________________________________________**

**Antibody Source Catalogue number**

**_______________________________________________________________________________________________**

**Primary antibodies (clone; host species):**

Anti-BrdU [BU1/75(ICR1); rat] Novus Biologicals NB500-169

Anti-CD3 (n/a; rabbit) Dako A0452

Anti-CD20cy (L26; mouse) Dako M0755

Anti-CD163 (10D6; mouse) Novus Biologicals NB110-59935SS

Anti-CD206 (n/a; rabbit) Millipore Sigma HPA004114

Anti-Dextran (DX1; mouse) Stemcell 60026

Anti-HAM56 (HAM56; mouse) ThermoFisher, eBioscience 14-6548-93

**Secondary antibodies (conjugate):**

Anti-chicken IgY (Alexa 488) ThermoFisher A-11039

Anti-mouse IgM (Alexa 488) ThermoFisher A-21042

Anti-mouse IgG1 (Alexa 488) ThermoFisher A-21121

Anti-mouse IgG1 (Alexa 568) ThermoFisher A-21124

Anti-mouse IgG (Alexa 568) ThermoFisher A11031

Anti-rabbit IgG (Alexa 488) ThermoFisher A-11008

Anti-rabbit IgG (Alexa 568) ThermoFisher A-11011

Ant-rat IgG (Alexa 568) ThermoFisher A-11077

n/a; not applicable, polyclonal antibody
