# Supplementary material for: SIV infection induces aging-like alterations in cardiac cellularity and macrophage populations of rhesus macaques
Source: J Leukoc Biol. 2026 Jun 5;118(6):qiag069. doi: 10.1093/jleuko/qiag069 (PMC13316664; doi:10.1093/jleuko/qiag069)
Supplement: qiag069_Supplementary_Data [file qiag069_supplementary_data.zip › Petkov et al Supplemental Figure legends.docx]

**Supplement Figure 1. Percentages of T and B lymphocytes in heart tissues from uninfected, SIV-infected, rhesus macaques. A.** A representative immunofluorescence image of ventricle tissue (animal IT75, adult, infected with SIV for 603 days) demonstrates staining for CD20+ B cells (turquoise arrow), CD3+ T cells (red arrow) and DAPI-stained cell nuclei (blue). **B.** Percentages of B and T cells were counted in ventricles of rhesus macaques with heart tissue histopathology scores of 0 – 2 (normal to mild) from adult animals 2 – 14.5 years of age that were uninfected, infected with SIV. Mann-Whiteny U test was performed for each set of cells and was non-significant for CD3+ T cells and CD20+ B cells.
