# Supplementary figures and images for: SIV infection induces aging-like alterations in cardiac cellularity and macrophage populations of rhesus macaques
Source: J Leukoc Biol. 2026 Jun 5;118(6):qiag069. doi: 10.1093/jleuko/qiag069 (PMC13316664; doi:10.1093/jleuko/qiag069)

Supplement 1 Figure

A

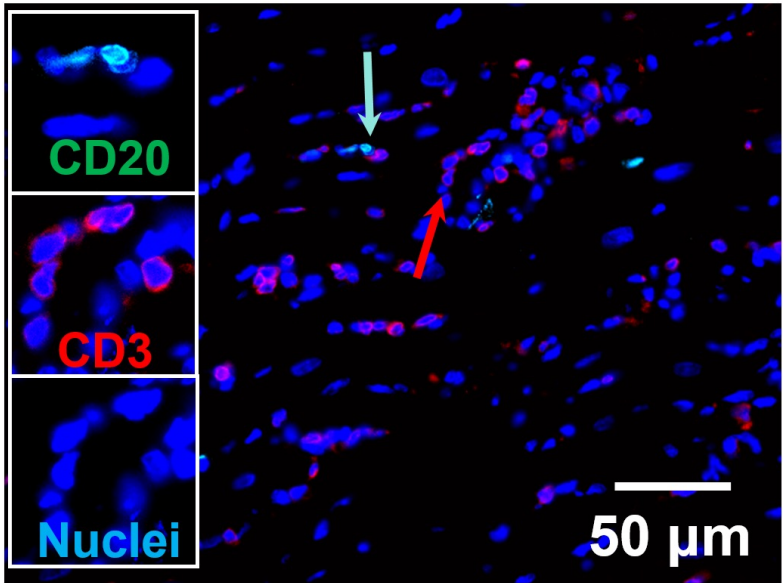

B

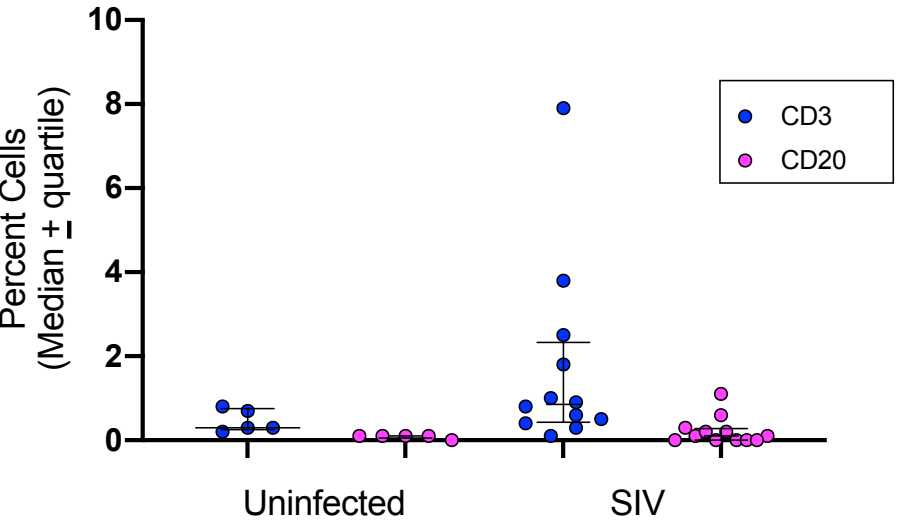

Supplement: qiag069_Supplementary_Data [file qiag069_supplementary_data.zip › Supplement figure 1 (1) updated.pdf]
